# Development of Flood Preparedness Behavior Scale: A Methodological Validity and Reliability Study

**DOI:** 10.1017/S1049023X24000189

**Published:** 2024-04

**Authors:** Marwa Osman, Gülşen Taşdelen Teker, Kerim Hakan Altıntaş

**Affiliations:** 1. University of Khartoum, Faculty of Medicine, Department of Community Medicine, Khartoum, Sudan; 2. Hacettepe University, Institute of Health Sciences, Department of Public Health, Ankara, Turkey; 3. Hacettepe University, Faculty of Medicine, Department of Medical Education and Informatics, Ankara, Turkey; 4. Hacettepe University, Faculty of Medicine, Department of Public Health, Ankara, Turkey

**Keywords:** behavior, flood, reliability, scale, validity

## Abstract

**Background:**

Floods are the most frequent natural disasters with a significant share of their mortality. Preparedness is capable of decreasing the mortality of floods by at least 50%. This paper aims to present the psychometric properties of a scale developed to evaluate the behavior of preparedness to floods in Sudan and similar settings.

**Methods:**

In this methodological scale development study, experts assessed the content validity of the items of the developed scale. Data were collected from key persons of 413 households living in neighborhoods affected by the 2018 floods in Kassala City in Sudan. A pre-tested questionnaire of sociodemographic data and the Flood Preparedness Behavior Scale (FPBS) were distributed to the participants’ houses and recollected. Construct validity of the scale was checked using exploratory factor analysis (EFA) and confirmatory factor analysis (CFA). Internal consistency of the scale was checked using Cronbach’s alpha. Test-retest reliability was assessed by Pearson’s correlation coefficient. Item analyses and tests of significance of the difference in the mean scores of the highest and lowest score groups were carried out to ensure discriminatory power of the scale items.

**Results:**

Experts agreed on the scale items. Construct validity of the scale was achieved using EFA by removing 34 items and retaining 25 items that were structured in three factors, named as: measures to be done before, during, and after a flood. Confirmatory factor analysis confirmed the construct obtained by EFA. The loadings of the items on their factors in both EFA and CFA were all > 0.3 with significant associations and acceptable fit indices obtained from CFA. The three factors were found to be reliable in terms of internal consistency (Cronbach’s alpha coefficients for all factors were > 0.7) and test-retest reliability coefficient. In item analysis, the corrected total item correlations for all the items were > 0.3, and significant differences in the means of the highest and lowest score groups indicated good item discrimination power.

**Conclusion:**

The developed 25 items scale is an instrument which produces valid and reliable measures of preparedness behavior for floods in Sudan and similar settings.

## Introduction

Flooding is the most prevalent natural disaster world-wide and has had multiple devastating impacts. It accounted for 47% of all the extreme weather event disasters in the world between the years 1969-2018.^1^ It accounted for 45% of all the nine natural disaster types in 2022, followed by storms, which accounted for 28%. The same year witnessed a huge flooding disaster in Pakistan that affected 33 million people and resulted in 1,739 deaths in addition to 15 billion US dollars in economic losses.^2^ Floods are increasing in frequency and intensity due to increase in their risk drivers that include climate change, environmental degradation, poor urban planning, poverty and inequity, and weak governance.^3^ Floods result in a number of health impacts including deaths, injuries, compromised hygiene both at personal and environmental levels due to destruction of latrines, transmission of communicable diseases and outbreaks, in addition to interruption of services and psychological effects.^4–6^ Socioeconomic impacts include destruction of houses, displacement, poverty, and inequality.^7–9^

Flooding disasters occur in Sudan almost every year.^10^ The largest flood disaster during this decade occurred in 2020, in which all the 18 states of the country were affected. The number of affected people were 880,000 in addition to 150 deaths and 175,000 destroyed or damaged houses.^10,11^ What makes the situations worse in Sudan is the absence of infrastructure and sewerage systems almost all over the country.^12^ The country is also liable to urban floods even in light rainfalls, due to poor urban drainage and low soil infiltration. The water lacks the drainages and remains in hollows formed by uneven land, stagnates, becomes breeding sites for the mosquitoes, and is only dried by the effect of the sun.^13^

Preparedness is one of the important public health measures that is effective in decreasing floods’ negative impacts. Preparedness is the knowledge and capacities that enable governments, communities, organizations, and individuals to effectively anticipate, respond to, and recover from the impacts of disasters.^14^ Preparedness includes measures to be taken before, during, and after the occurrence of floods. It decreases the mortality of floods by at least 50%.^15^ It is the key factor that determines the effectiveness of early warning systems and was associated with a decrease in flooding losses.^16^ Increasing the preparedness of individuals for floods necessitates providing knowledge, improving the attitudes and competencies, and assessing them. Measuring preparedness is essential for making decisions about problematic areas in preparedness to improve them.

The tools available in the literature for measuring flood or disaster preparedness either focus on measuring the attitude, focus on the emergency kit over the other indicators, target a special group like nurses and not the general population, or are not suitable for use in Arabic speaking low-income country like Sudan.^17–20^

This study aims to develop a scale that measure the individual’s behavior of preparedness to floods and provide a preliminary analysis of the scale’s psychometric properties of validity and reliability. This scale is beneficial for individual assessment of self-behavior towards preparedness and provides a tool for researchers to assess flood preparedness in surveys and preparedness campaigns. This scale provides a quantitative description of the level of preparedness of individuals and allows comparisons of these levels.

## Methods

### Study Design and Setting

This is a methodological validity and reliability study. Steps of this study were carried out from 2020-2022. The validation study was carried out in Kassala City in eastern Sudan. The city has a known history of floods in the Gash River and other seasonal watercourses. In 2003, the floods of the Gash River washed two-thirds of the city and floods continued to occur in less intensity in a rate estimated by once every five years.^21^ In 2018, Kassala City was affected by massive floods of the Emiray seasonal watercourse that resulted in death, displacement, and thousands of affected people.

### Item Development

A systematic review of the literature was conducted for the scales that measure flood or general disaster preparedness among the general population.^22^ In addition, the guidelines of different countries for preparing their populations for floods were reviewed, including the awareness materials of Ministry of Health of Sudan (Khartoum, Sudan). Most of these guidelines divide preparedness to measures to be done before, during, and after floods.

An item pool of 58 relevant items was developed. A five-point Likert scale of “I always do that,” “most of the time,” “sometimes,” “rarely,” and “I never do that” was selected as a response format. An additional response option was added which is “not applied to me.” This option should be selected when the questions are not relevant to the respondents; for example, questions about driving during floods which are not relevant to those who do not drive or use a car.

The item topics were: following the source of information; receiving early warning; following the expected level of rain in the area of residence, and in the upstream area; choosing the place of living according to risk of floods; using water resistant building constituents; speaking with family about preparedness; taking measures to ensure water drainage from the house; preparing a plan of evacuation; preparing plans to evacuate those who need; preparing safe place; preparing safe place for animals and livestock; testing effectiveness of the evacuation plan; preparing an emergency kit; discussing preparedness with community; participating with community in preparedness activities; having flood insurance; evacuating a building when feeling it will collapse; taking the emergency kit when evacuating; turning off valves of electricity, water, and gas before evacuation; conducting effective evacuation and saving self, family, animals, documents, and important belongings; avoiding to walk in flood water; refrain swimming in flood water; avoiding to drive through flood water; items related to what to do if trapped in a building or a vehicle (three items); finding access to safe drinking water after flood; purifying water; finding access to safe latrines after flood; what to do in absence of latrines (two items); avoiding driving through flood water after the flood; returning to flooded area only when authorities state that returning is safe; checking for loose boards and slippery floors when entering a flooded building; turn off electricity when entering; keeping eye on dangerous debris; wearing personal protective equipment; staying away from powerlines, electrical poles; reporting downed power lines to the electricity company; refrain from touching wet electrical equipment; refrain from touching it while standing on water; using stove or generators outdoors and away from windows; applying anti-vector measures for self, and for family; fixing damaged toilets or septic tanks; making sure that drinking water is checked for contaminants; clean everything that gets wet during flood; disinfect what needs disinfection; throwing wet food after flood; and providing psychological support to needy.

### Procedure

To check for content validity of the developed item pool, it was subjected to thorough review by a panel of seven experts. Four of them had expertise in disaster management and environmental health, and one expert was from the field of scale development, medical education, and educational evaluation. In addition, two language experts, one in English and one in Arabic, contributed to the process of scale development to ensure language validity (Table 1). The content validity index (CVI) was calculated for every item in terms of representativeness and clarity, as recommended by Rubio, et al, from the public health experts’ reports.^23^ A four-point scale of “relevant and clear, needs no change,” “needs minor changes,” “needs major changes,” and “not relevant and should be omitted” was used. These experts were also asked to add items wherever needed. The outcome of this procedure was that all items had a CVI of 1.0 and one item about applying anti-rodent measures was added to the scale to make up a draft scale of 59 items. A pilot study was conducted in the target population. Five men and five women responded to the scale directly, without questions, and stated that the items of the scale were clear.

**Table 1.** Characteristics of Panel Experts

Expert Number	Qualification and Role in Panel	Age at Time of Consultation	Gender	Area of Expertise	Years of Experience
Exp. No: 1	Professor, MD, content validity consultant	69	Male	Public health, Environmental health, Disaster public health	40
Exp. No: 2	Professor, MD, content validity consultant	70	Male	Public health, Health promotion, Disaster public health	41
Exp. No: 3	Professor, MD, content validity consultant	53	Male	Public health, Communicable disease control, Disaster management	25
Exp. No: 4	Professor, MD, content validity consultant	44	Male	Disaster management, Public health	13
Exp. No: 5	Professor, PhD, scale development consultant	45	Male	Educational assessment and evaluation	17
Exp. No: 6	Language consultant	26	Female	English literature (native speaker)	4
Exp. No: 7	Language consultant	37	Male	Arabic Journalism editing (native speaker)	14

### Participants

Participants of this study were 413 household key persons living in the neighborhoods affected by the 2018 floods in Kassala City in Sudan. This sample size accounted for 16% of the total number of households affected by flooding in Kassala in 2018. The sample size was determined as 400 to fulfil the criteria of the sample size required to perform factor analyses, and to ensure heterogeneity of the sample required in validation studies.^24–26^

### Data Collection

Sociodemographic data questionnaire and Flood Preparedness Behavior Scale (FPBS) were distributed to the 413 households in the seven neighborhoods (50-70 questionnaire for each neighborhood). Data collection was carried out by the help of two to three of the local people from each neighborhood who delivered the questionnaires to the respondents’ houses and recollected them after filling. This process was carried out under the supervision of the researcher. These local people were told not to interfere in the filling process of the questionnaires and let the respondents answer just the way they understand the questions. Questionnaires were distributed using a convenience sampling method. According to Osman, et al,^22^ this method was used in 10 out of 12 scales included in the systematic review of the literature for the scales measuring disaster preparedness among the general population.^17,19,27–34^ This is also consistent with the recommendations of Clark, et al regarding the first administration of a newly developed scale that can be carried out among a sample of convenience of 100-200 individuals. Clark, et al also recommend that the subsequent steps of validation to be carried out among a heterogenous sample of more than 300 individuals.^24^

### Data Analysis

Validity is defined in this research as the extent to which the scale measures what it is supposed to measure, which implies extent to which preparedness behavior variable is the underline cause of item covariation.^26^ Content validity (as explained above) and construct validity were evaluated for FPBS. Construct validity was assessed using exploratory factor analysis (EFA) and confirmatory factor analysis (CFA). It is recommended to carry out EFA and CFA on different datasets. Therefore, data were randomly divided into two datasets of 230 and 183 for the EFA and CFA, respectively. A sample size of 150-200 is considered adequate to enable carrying out factor analyses.^35^ Data were found to be appropriate for carrying out factor analysis as per the three criteria: (1) adequate sample size, (2) Kaiser-Meyer-Olkin (KMO) value obtained from EFA, and (3) Bartlett’s test which was found statistically significant.^35,36^ Results obtained from EFA were also checked using parallel analysis and CFA. Reliability is defined in this research as the extent to which the scale performs in consistent, predictable ways, which implies the extent to which the scores it yields represent the true state of individual preparedness behavior. Internal consistency (by Cronbach’s alpha), composite reliability (calculated using the formulas provided by Fornell and Larcker^37^), and test-retest reliability coefficients were estimated for the measures obtained from the scale. In addition, item analysis, including corrected item total correlations for every item, was calculated to examine the items’ power of discrimination. Comparisons of mean scores of the 27% highest and lowest groups were carried out as well.

### Ethical Approval

Ethical approval for this study was obtained from the Non-Interventional Research Ethics Committee of Hacettepe University (Ankara, Turkey; Decision Number 2020/01- 05). A written informed consent was obtained from every participant.

## Results

### Sociodemographic Characteristics of the Sample

Females were 58% of the sample. The range of age was from 18 through 76. The mean (SD) and the median (1^st^ -3^rd^ Quartiles) for the age of the participants were 30.45 (SD = 11.64) and 28 (22 - 35.75), respectively. The educational level of the participants from the highest to the lowest percentage was as follows: secondary school graduates (38.9%), primary school graduates (28.2%), university graduates (19.7%), literate with no certificates (9.5%), and those with postgraduate degrees (3.7%). The sample included 46.1% married respondents and 47.3% single, in addition to 6.6% widowed or separated from the spouse. A total of 49.0% of the participants had children. They had diverse jobs. These characteristics fulfilled the criteria for heterogeneity required in validation studies.

### Construct Validity of the Behavior Scale

#### Findings of the EFA of the FPBS

Exploratory factor analysis was performed for the behavioral scale and resulted in removal of 34 and retention of 25 items. Items were removed if they loaded into two factors or loaded on the factor by less than 0.3. The KMO value of sample adequacy of model of the scale with 25 items out of 59 items was 0.850 (>0.6), and the Bartlett test was highly significant (P value less than .001). This indicated that the sample was suitable for carrying out factor analysis.^35,36^ The remaining 25 items were structured into three factors named as: measures to be carried out before, during, and after the flood. These factors included seven, seven, and eleven items, respectively. The percentage of variance explained by this model was 40.95%.

The loadings of the first seven items on the first factor ranged from 0.42-0.70. Loadings of the second seven items on the second factor ranged from 0.52-0.74. In addition, the loadings of the final eleven items of the scale on the third factor ranged from 0.35-0.73. The items of the three factors and their factor loadings obtained from EFA using varimax rotation are explained in Table 2.

**Table 2.** FPBS Factor Structure and Factor Loadings (n = 230)

Item	Factor 1 (Before)	Factor 2 (During)	Factor 3 (After)
1- B2- I receive early warning messages about the occurring floods.	.538		
2- B4- During the flood season, I follow up the level of rains in the upstream areas.	.426		
3- B9- I prepare a plan of evacuation of the flooded place to a safe place.	.602		
4- B10- We prepare special plans to evacuate people who need help during evacuation (eg, those with disabilities, with special needs, elderly people, and children).	.611		
5- B11- I prepare a safe place where I can go with my family enough time before the onset of floods.	.701		
6- B13- I test the effectiveness of our evacuation plan by practicing it with my family.	.677		
7- B14- I prepare an emergency kit in which I store important things to quickly grab when evacuation is needed.	.693		
8- B19- I immediately evacuate the building I am in, if I feel it will collapse because of the flood.		.596	
9- B21- I turn off gas, electricity, and water before evacuating a flooding place.		.671	
10- B22- If the situation requires it, I can conduct the evacuation effectively and save myself.		.654	
11- B23- If the situation requires it, I effectively evacuate and save my family.		.741	
12- B25- If the situation requires it, I effectively evacuate and save my important documents.		.601	
13- B27- I avoid walking through floodwaters.		.522	
14- B28- I refrain from swimming through flood waters.		.542	
15- B41- I check for loose walls and slippery ﬂoors when entering a flooded home or building.			.520
16- B42- I turn off electricity at the main breaker or fuse box when entering a flooded building.			.495
17- B47- I report downed power lines immediately to power company’s emergency number.			.736
18- B48- I refrain from touching electrical equipment if it is wet.			.352
19- B49- I refrain from touching an electrical equipment while standing in water.			.618
20- B54- I immediately fix the septic tanks and the toilets damaged because of floods as soon as possible.			.535
21- B55- We ensure that our wells and drinking water are checked for bacterial and chemicals contamination after flooding.			.451
22- B56- I clean everything that gets wet with floodwater.			.570
23- B57- I disinfect everything that requires disinfection, if it becomes wet during a flood.			.646
24- B58- I throw any food that gets wet by floods’ water.			.548
25- B59- I suggest ways to provide psychological support to those who need it after floods.			.541

Abbreviation: FPBS, Flood Preparedness Behavior Scale.

**Figure 1.**
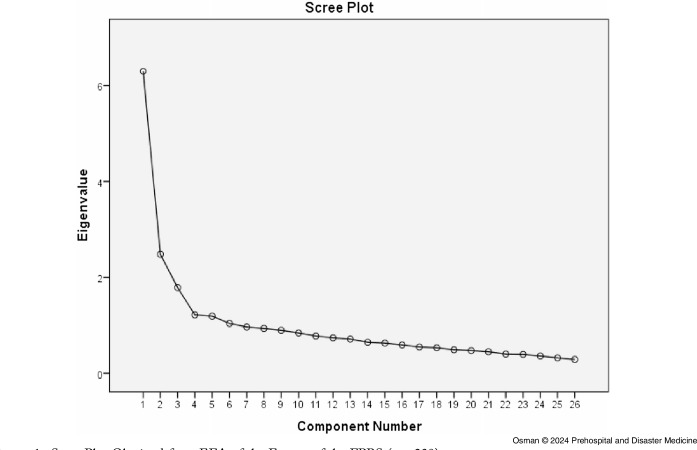
Scree Plot Obtained from EFA of the Factors of the FPBS (n = 230). Abbreviations: EFA, exploratory factor analysis; FPBS, Flood Preparedness Behavior Scale.

The scree plot obtained from EFA, which is the plot of the eigenvalues of all the factors, supported the model of three factors (Figure 1). It showed a sharp descending line between factor one, two, and three and an almost horizontal line for the other factors. This supports the three-factor model of the scale. The scree plot is presented in Figure 1.

In addition, parallel analysis also supported three factor structures of the behavioral scale, using 250 cases of the sample for the 25 variables of the FPBS. Parallel analysis compares the real eigenvalues to those estimated using random data. Each one of the real eigenvalues of the first three factors which were 6.101, 2.399, and 1.738, respectively, was greater than the corresponding eigenvalue estimated from random data which were 1.6207, 1.5232, and 1.4532. The opposite was the case for the fourth factor, where the eigenvalue obtained from the random data (1.3845) was greater than the real eigenvalue (1.207). This is the gold standard and the most accurate method in determining the number of factors of a scale.^35^ Three factors were determined based on these methods.

#### CFA Findings of FPBS

**Figure 2.**
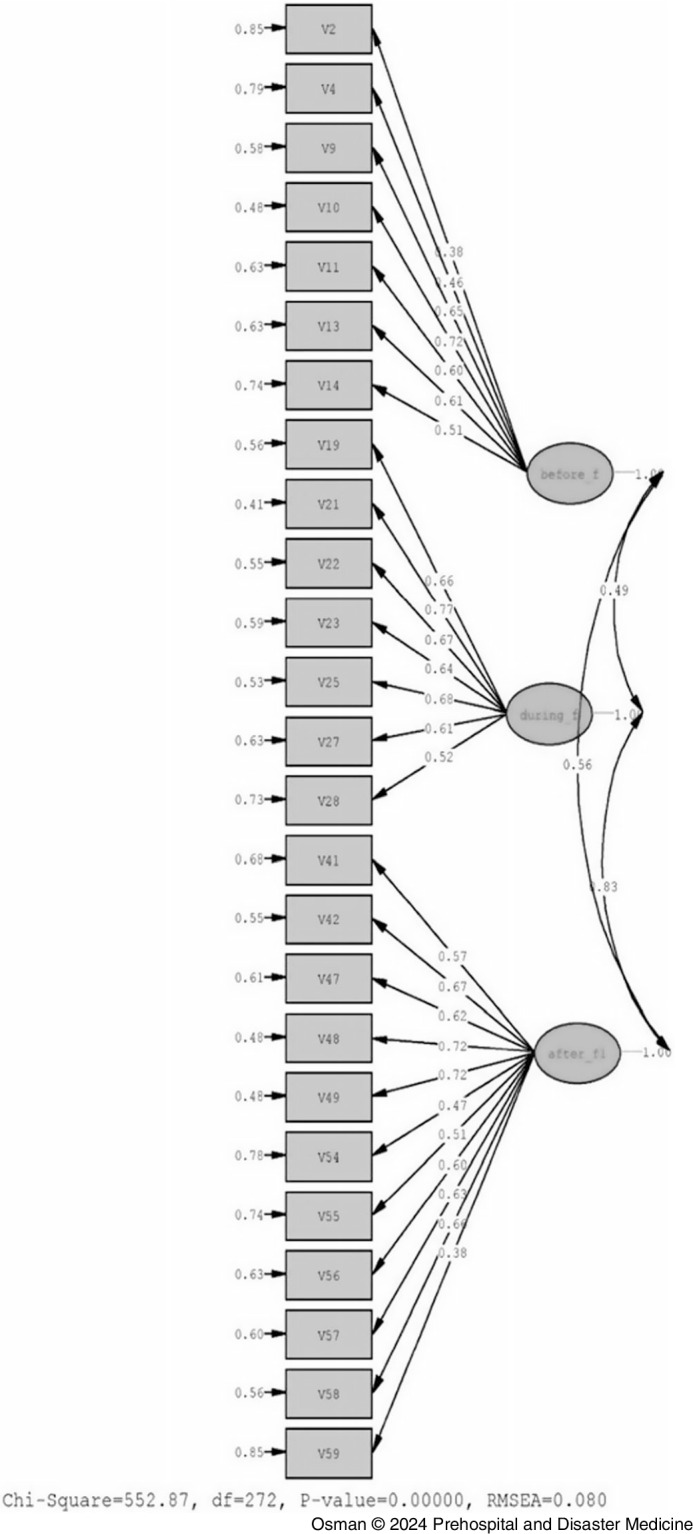
Factor Loadings Obtained from CFA of the Items of the FPBS (n = 183). Abbreviations: CFA, confirmatory factor analysis; FPBS, Flood Preparedness Behavior Scale; χ2, Chi square; df, degrees of freedom; RMSEA, root mean square error of approximation.

Loadings of the items on the factors obtained from CFA are illustrated in Figure 2. The range of item loadings on the first, second, and third factors were between 0.38-0.72, 0.52-0.77, and 0.38-0.72, respectively. According to these values, no item was loading by less than 0.3, and therefore, no item needed to be removed from the model.

The obtained t values of the model for the items of the first, second, and the third factors ranged from 4.57- 9.57, 6.69-11.01, and 4.79- 10.22 for the three factors, respectively. All items were loading significantly on their factors at P value of .05 and .01 as all the t values were greater than both 1.96 and 2.58. This indicated that the estimations of factor loadings were significant and no item needed to be removed from the model.

Statistics of goodness of fit obtained from the CFA of the FPBS revealed acceptable values for the indices Chi square (χ2)/degrees of freedom (df), comparative fit index (CFI), non-normed fit index (NNFI), incremental fit index (IFI), root mean square error of approximation (RMSEA), standardized root mean square residual (SRMR), parsimony normed fit index (PNFI), and parsimony goodness of fit index (PGFI). The fit indices for the three-factor structure of the behavior scale are presented in Table 3.

**Table 3.** Fit Indices of FPBS Obtained from CFA Compared to Criteria of Perfect and Acceptable Fit (n = 183)

Examined Index	Perfect Fit Value	Acceptable Fit Value	Obtained Value	Result
χ^2^/df	0 ≤ χ^2^/df ≤ 2	2 ≤ χ^2^/df ≤ 3	2.033	Acceptable
CFI	.95 ≤ CFI ≤ 1.00	.90 ≤ CFI ≤ .95	0.93	Acceptable
IFI	.95 ≤ IFI ≤ 1.00	90 ≤ IFI ≤ .95	0.93	Acceptable
RMSEA	.00 ≤ RMSEA ≤ .05	90 ≤ IFI ≤ .95	0.08	Acceptable
SRMR	00 ≤ SRMR ≤ .05	.05 ≤ SRMR ≤ .10	0.082	Acceptable
PNFI	95 ≤ PNFI ≤ 1.00	.50 ≤ PNFI ≤ .95	0.80	Acceptable
PGFI	95 ≤ PGFI ≤ 1.00	.50 ≤ PGFI ≤ .95	0.66	Acceptable

Abbreviations: CFA, confirmatory factor analysis; FPBS, Flood Preparedness Behavior Scale; χ2, Chi square; df, degrees of freedom; CFI, comparative fit index; IFI, incremental fit index; RMSEA, root mean square error of approximation; SRMR, standardized root mean square residual; PNFI, parsimony normed fit index; PGFI, parsimony goodness of fit index.

#### Reliability Analyses Findings of the FPBS

The reliability evidence of FPBS was obtained by calculating three reliability coefficients. First, the internal consistency was estimated by calculating Cronbach’s alpha, which was found 0.80, 0,81, and 0.86 for the three factors, respectively. Second, composite reliability coefficients were found 0.77, 0.84, and 0.86, respectively, for the three factors. Finally, to prove temporal stability, the scale was applied twice at two-week intervals to 31 participant and correlation coefficients for the test and retest scores were calculated. The test-retest reliability coefficient was 0.63 for the scale, which indicated a moderate temporal stability of the scale.

#### Item Analysis for Behavior Scale

Table 4 presents the results of the FPBS item analysis. All the total corrected item correlations were greater than 0.3, which indicated that the items were sufficiently discriminant. The values of Cronbach’s alpha if an item was deleted for every item was less than the obtained Cronbach’s alpha for almost all the items.

**Table 4.** Item Analysis for the Items of FPBS (n = 413)

Factor	Item	Corrected Total Item Correlation	Cronbach Alpha if Item Deleted	Mean Score of Item	Standard Deviation	Skewness
Before Flooding	B2	0.463	0.786	2.92	1.335	0.245
	B4	0.440	0.790	3.25	1.458	−0.139
	B9	0.535	0.773	3.56	1.488	−0.496
	B10	0.563	0.770	3.83	1.357	−0.797
	B11	0.601	0.761	3.58	1.530	−0.593
	B13	0.587	0.764	3.04	1.512	−0.057
	B14	0.536	0.774	3.07	1.603	−0.016
During Flooding	B19	0.501	0.795	4.14	1.371	−1.334
	B21	0.620	0.773	4.26	1.201	−1.538
	B22	0.621	0.772	4.16	1.221	−1.361
	B23	0.613	0.777	4.43	1.067	−1.966
	B25	0.614	0.774	4.22	1.262	−1.481
	B27	0.491	0.795	4.27	1.194	−1.512
	B28	0.405	0.813	4.14	1.411	−1.413
After Flooding	B41	0.486	0.856	4.28	1.167	−1.536
	B42	0.485	0.855	4.37	1.177	−1.829
	B47	0.729	0.836	4.18	1.289	−1.304
	B48	0.681	0.841	4.45	1.122	−2.062
	B49	0.695	0.840	4.48	1.108	−2.115
	B54	0.490	0.855	3.95	1.279	−0.937
	B55	0.452	0.860	3.79	1.406	−0.816
	B56	0.515	0.853	4.41	1.072	−1.072
	B57	0.676	0.843	4.35	1.110	1.110
	B58	0.551	0.852	4.59	0.912	1.302
	B59	0.445	0.860	4.00	−2.373	−1.030

Abbreviation: FPBS, Flood Preparedness Behavior Scale.

Analyses were carried out for differences in the means between the two groups of the highest and lowest 27%, according to the total scores of the three factors. These means of the highest and lowest scores for the three factors were: 31.76-15.47, 34.74-23.71, and 54.25-37.39, respectively. The differences between these means were significant, with a P value of less than .01. This finding supports the discrimination power of the items.

#### Interpretation of the FPBS

The used five-point scale that ranged from five (I always do that) to one (I never do that) in the FPBS made the total score of the 25 items range between 25 and 125. High scores with a maximum value of 125 indicated perfect practice, and low scores with a minimum value of 25 indicated poor practice. There was no need to reverse items in the scale, as there were no negative items in the FPBS.

## Discussion

The aim of this study is to provide a valid and reliable scale to measure the appropriateness of the flood preparedness behavior among the general population. A panel of experts reviewed the developed item pool to achieve the objectives of the study. This check of content validity is considered the most important step in developing a new scale.^38^ Exploratory factor analysis of the data revealed structuring of the items in three factors named as measures to be done before, during, and after the flood. This is consistent with the guidelines of flood preparedness issued for individual preparedness in different countries.^39–41^ The total variance explained by this three-factor structure was 40.95%, which is considered acceptable.^42,43^ All the items loaded on their factors by more than minimum level of 0.3.^35^ Analyses revealed acceptable CFA fit indices, acceptable Cronbach alpha and test-retest reliability indices, and item discrimination power.^38,44–47^

The final scale included 25 items and excluded 34. The topics of most of the included items were also included in the items of previous general disaster or earthquake preparedness measuring scales, or the sole attitude measuring flood preparedness scale.^26^ The first item’s topic was receiving early warning messages. This topic was also included in four previously published scales, including the sole flood scale.^17,31,32,48^ The second item is related to following up the level of rain in the upstream area of a river, which was found effective in Kassala in the 2007 floods.^21^ This is also beneficial in many parts of Sudan and in similar settings of rivers and seasonal watercourses. Items related to evacuation plans in consistency with their inclusion in this scale were included in seven previous scales.^17,27–31,48^ Items related to the emergency kit were also included in five scales.^17,29–31,48^ Items related to turning off valves of electricity, water, and gas were included in three previous scales.^27,28,31^ Items related to loose walls, collapse of building, and powerlines and power poles are of special importance in Sudan as most of the deaths from floods in Sudan are related to them.^49^ Number of damaged septic tanks or latrines is an important indicator of the magnitude of flooding in Sudan. The items related to checking of the drinking water, throwing food, and cleaning and disinfection of objects are important post-flooding measures to decrease diseases that can occur, especially in unsewered areas. An item related to psychological support was also included in the scale of Inal, et al.^29^ Thus, the included items were specific and inclusive of the important items regarding flood preparedness. These included items were also consistent with the items recommended in all hazard approach of disaster preparedness, as they include the triad of having a kit, preparing a plan, and staying informed.^50,51^ They are also consistent to the top hazard approach of disaster management being specific to floods.^52^ On the other hand, the items which were not included were either included in their meaning in another item or were not specific to preparedness for floods. For example, following up the source of information is included in its meaning in receiving an early warning, and items related to applying anti-vector and anti-rodent measures are not specific measures to flooding preparedness; they mainly belong to epidemic and disease control measures.

The option “not applicable to me” was put in this scale during the validation study and was agreed upon by the experts. Putting such an option increases the validity of the scale because it measures what it is actually supposed to measure. This option was analyzed as missing, and most of the responses of that option were the items related to the cars or vehicle driving, which were already removed by EFA. Thus, there was no need to include this option in the final version of the scale, as all its items are applicable to every adult individual.

This scale will help to quantitatively measure flooding preparedness. It can be used by individuals to measure their own preparedness behavior, as well as by researchers in surveys carried out to evaluate flood preparedness. It can also help in directing awareness campaigns towards areas of poor behavior and can serve as a tool for evaluating these campaigns. This scale can improve the decisions that can be made in order to increase the levels of preparedness to floods.

## Limitations

This validation study did not include checking for criterion validity, as this is the only scale that measures flooding preparedness practice in the literature. The validation study similar to most of the other validation studies was carried out in one city. Further validation studies of this scale in different settings can improve the scale and empower the evidence of its validity and reliability.

## Conclusion

The scale of 25 items was found to be a valid tool to measure individual flooding preparedness behavior, as per the findings of content validity indices, EFA, and CFA. The scale was found to be internally consistent with the findings of Cronbach’s alpha of above 0.70 and reliable as per the findings of composite reliability and test-retest correlation coefficient. The items of the scale were found to have acceptable power of discrimination according to the corrected total item correlations and the significant difference in means of scores of the 27% highest and lowest score groups. These findings support the hypothesis that this scale is an instrument that produces valid and reliable measures of individual preparedness for flooding in Sudan and similar countries.
